# Bone marrow mesenchymal stem cells improve thymus and spleen function of aging rats through affecting P21/PCNA and suppressing oxidative stress

**DOI:** 10.18632/aging.103186

**Published:** 2020-06-19

**Authors:** Zhihong Wang, Yun Lin, Shang Jin, Tiannan Wei, Zhihai Zheng, Weimin Chen

**Affiliations:** 1 Provincial Clinical Medical College of Fujian Medical University, Fuzhou 350001, Fujian, China; 2 Department of Hematology, Fujian Provincial Hospital, Fuzhou 350001, Fujian, China

**Keywords:** BMSCs, immune system, aging, P21/PCNA, oxidative stress

## Abstract

Bone marrow mesenchymal stem cells (BMSCs) have been considered to be an important regulator for immune function. We aim to prove the function improvement of aging spleen and thymus induced by BMSCs and unfold the specific mechanisms. Aging animal model was established using D-galactose. The morphological changes of spleen and thymus tissues were observed using hematoxylin-eosin staining and transmission electron microscopy. Key cytokines in the serum were measured with enzyme linked immunosorbent assay. Protein and mRNA levels of P16, P21, and PCNA were detected using western blotting and RT-qPCR. Special markers of BMSCs were identified using flow cytometry, and successful induction of BMSCs to steatoblast and osteoblasts was observed. Compared to aging model, BMSCs significantly increased the spleen and thymus index, improved the histological changes of spleen and thymus tissues. A remarkable increase of ratio between CD4+T cells and CD8+T cells, level of IL-2 was achieved by BMSCs. However, BMSCs markedly inhibited the content of IL-10, TNF-*a*, P16, and P21 but promoted PCNA. Significant inhibition of oxidative stress by BMSCs was also observed. We demonstrated that BMSCs significantly improved the tissue damage of aging spleen and thymus, BMSCs may improve aging organs through influencing cytokines, oxidative stress, and P21/PCNA.

## INTRODUCTION

Dysfunction of immune system is an important symbol for aging body. Thymus is a central lymphoid organ responsible for production of naive T cells, which plays a vital role in mediating both cellular and humoral immunity [1]. Chronic involution of thymus gland is thought to be one of the major contributing factors to immune function loss with increasing age [2]. Spleen is commonly involved in the regulation of humoral immunity [3]. Both thymus and spleen have been considered to be closely linked with body aging.

Bone marrow mesenchymal stem cells (BMSCs) are stem cells with strong proliferation ability and multidirectional differentiation potential [4]. BMSCs can differentiate into endothelium, muscle, nerve cells, and thymic matrix. BMSCs can be used as seed cells for cell therapy because of homing to damaged tissues, repairing tissues and regulating immune system [5, 6].

Aging process is closely associated with the increase of oxidative stress, the release of cytokines, and further dysregulation of immune system [7–10]. BMSCs have been proven to play a vital role in regulating inflammation and immune function [11, 12]. Nowadays, decrease of stem cells is believed to be one of aging mechanisms. Meanwhile, BMSCs have presented an application potential in ischemic stroke [13] and lung injury induced by pneumonia [14] and traumatic brain injury [15]. However, if BMSCs could improve the function of aging thymus and spleen and the specific mechanism are not fully clear. PCNA and P21 have been believed to be closely linked with DNA replication and repair [16–19], whether BMSCs could affect the expression of PCNA and P21 has not been reported.

D-galactose has been widely used to establish aging animal model because of its ability to diminish immune response, decrease antioxidant enzyme activity, and increase reactive oxygen species (ROS) level [20–23]. In this study, the aging rat model was established using D-galactose. We further confirmed the effects of BMSCs transplantation on cytokines and targeting molecules linked with oxidative stress.

In the present study, we successfully proved the improvement of both structure and immune function of aging thymus and spleen tissues after treatment with BMSCs. Meanwhile, part of potential mechanism might be related with oxidative stress, DNA repair, and regulation of cytokines, P16, P21, and PCNA. This study unfolds the potential application of BMSCs and provides novel insight aiming at improving aging.

## RESULTS

### Isolation, culture and identification of rat BMSCs

**Figure 1:**
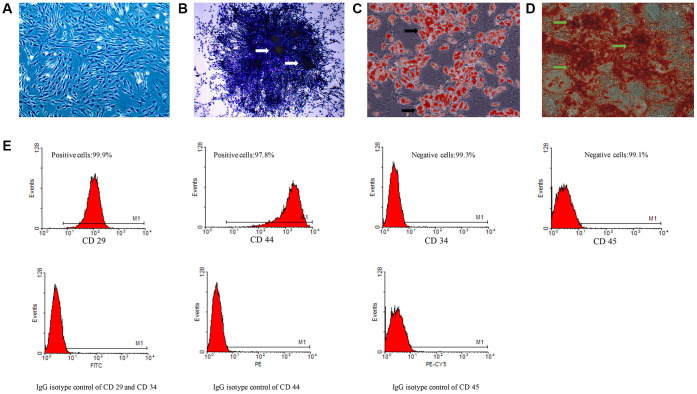
**The isolation and identification of BMSCs and directional differentiation of BMSCs to steatoblast and osteoblasts.** (**A**) The isolation and culture of BMSCs; (**B**) Clone formation ability of BMSCs stained by crystal violet staining; (**C**) Induction of steatoblast stained by Oil Red O staining; (**D**) Induction of osteoblasts stained by alizarin red staining; (**E**) Identification of surface markers of BMSCs by flow cytometry. White arrows indicate clone formation; Black arrows indicate lipid vesicles; Green arrows indicate calcified nodules.

After 3 passages, the cells are star-shaped and spindle-shaped on the view of light microscope. After another 3 consecutive passages, no significant changes in cell morphology were observed, indicating that BMSCs were purified (Figure 1A). Meanwhile, we observed the formation of clone ability of BMSCs (Figure 1B). To further identify the characteristic of BMSCs, we tested the differentiation ability of BMSCs. After incubation with adipogenic induction solution for 72 h, small lipid droplets concentrated around the nucleus. As the induction time prolonged, the lipid droplets gradually aggregated into large lipid vesicles (Figure 1C). After 21 days’ osteogenesis induction, the positive calcified nodule was clearly observed using alizarin red staining (Figure 1D). The biomarkers of BMSCs were measured by flow cytometry. 99.9% CD29 and 97.8% CD44 positive cells, 99.3% CD34 and 99.1% CD45 negative cells were observed suggesting that the cells we isolated should be BMSCs (Figure 1E).

### The location identification of green fluorescent protein (GFP)labeled BMSCs in the thymus and spleen tissues

**Figure 2:**
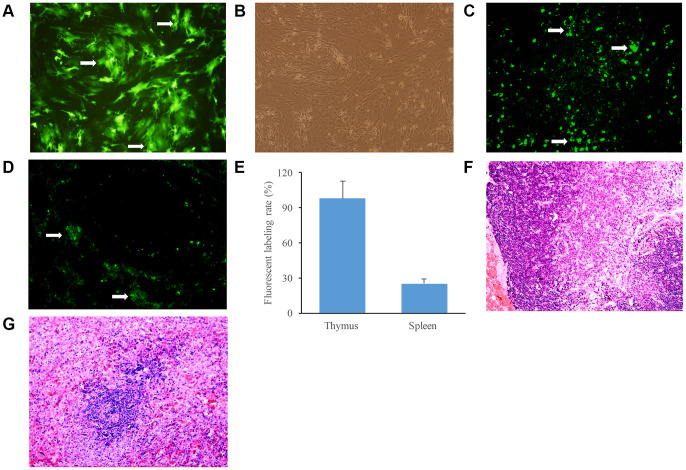
**Identification of GFP labeled BMSCs in the thymus and spleen tissues.** (**A**) BMSCs were observed after transfection with GFP for 24 h; (**B**) BMSCs in the same field were observed by ordinary inverted microscope; (**C**) GFP labeled BMSCs were observed in the thymus tissues of aging rats after infusion with BMSCs; (**D**) GFP labeled BMSCs were observed in the spleen tissues of aging rats after infusion with BMSCs; (**E**) The fluorescent labeling rate was measured after infusion with BMSCs; (**F**) The thymus tissues of aging rats were stained by HE staining; (**G**) The spleen tissues of aging rats were stained by HE staining. White arrows indicate green fluorescent cells.

After transfection with adenovirus carrying GFP for 24 h, strong green fluorescence was observed in the BMSCs, and the transfection efficiency was about 80% (Figure 2A). We then treated rats with GFP labeled BMSCs through caudal vein infusion. The obvious fluorescent cells were observed in both thymus and spleen tissues after 3 days (Figure 2C and 2D). Around 95% and 25% of fluorescent labeling rate were achieved after infusing BMSCs into thymus and spleen, respectively (Figure 2E). Normal thymus and spleen tissues were also investigated through hematoxylin-eosin (HE) staining (Figure 2F and 2G). These findings indicated that GFP labeled BMSCs had been successfully delivered to thymus and spleen tissues.

### BMSCs improved the morphological changes of thymus and spleen tissues of aging rats

**Figure 3:**
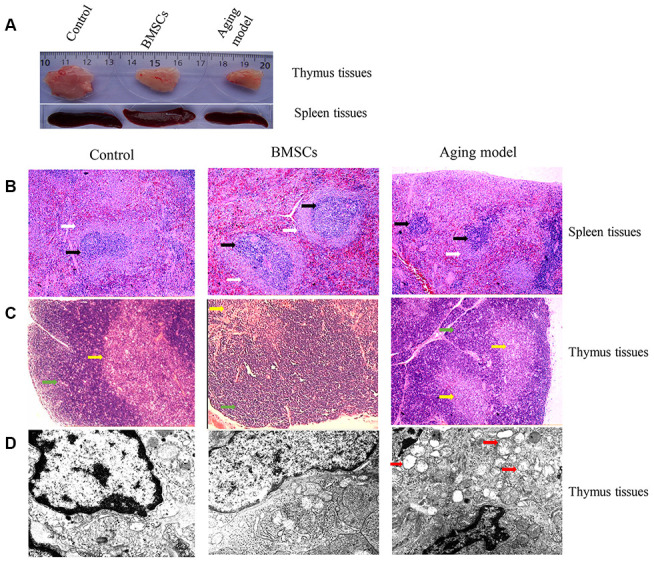
**BMSCs improved the morphological changes of thymus and spleen tissues of aging rats.** (**A**) Morphological changes of thymus and spleen tissues were observed through naked eyes; (**B**) Histological changes of spleen tissue were investigated after HE staining; (**C**) Histological changes of thymus tissue were investigated after HE staining; (**D**) Histological changes of thymus tissue was investigated using transmission electron microscopy. White arrows indicate white pulp; Black arrows indicate splenic nodule; Green arrows indicate cortex; Yellow arrows indicate medulla; Red arrows indicate vacuoles in the cytoplasm of epithelial reticular cells.

After treatment with BMSCs, the morphological changes of thymus and spleen tissues of aging rats were investigated. Significant differences in thymus and spleen tissue structure were observed in the three groups. Through the visual inspection, the size of thymus and spleen tissues were markedly decreased by the treatment of D-galactose, while, BMSCs could significantly reverse those influences (Figure 3A).

In the control group, we could observe that the size of spleen nodules was uniform, the boundary between white pulp and red pulp was clear, and the border area was obvious. However, for the spleen tissues in the aging model group, the proportion of white pulp in the spleen was relatively less, and the boundary between white pulp and splenic nodule was not clear, which conformed to the histological characteristics of aged spleen. After treatment with BMSCs, the size of splenic nodules was not consistent, but the white pulp area was enlarged and the histological changes of aging was improved compared with aging model group (Figure 3B).

In the control group, the thymic cortex was thicker, the medulla was smaller, the thymic lobule differentiated clearly and the boundary was clear. However, in the aging model group, thinner thymic cortex, bigger medulla, less thymic lobule, and unclear boundary were observed (Figure 3C). BMSCs treatment remarkably improved the morphological structure of thymus, the cortex and medulla of the thymic lobule were clearly defined, the thickness of the cortex was obviously increased, and the cell density was increased (Figure 3C).

We further investigated the histological changes of thymus tissue using transmission electron microscopy (TEM). In the control group, the thymic cortex cells were densely packed, the thymocyte boundaries were clear, and the ratio of nuclear and cytoplasm was moderate. However, in the aging model group, thymocytes were sparsely arranged, the periphery of the cells was blurred, pyknosis and apoptosis of some nucleus were observed. Meanwhile, more vacuoles in the cytoplasm of epithelial reticular cells, increased adipose tissue in the thymus, widened interlobular septum, increased connective tissue, and fibrous tissue hyperplasia was observed. Treatment with BMSCs remarkably improved the histological changes of thymus tissue compared with aging model group. The ultrastructure of thymocytes, epithelial reticular cells and macrophages were normal, and the intracellular organelles were rich and intact (Figure 3D).

### Influence of BMSCs on the transformation function of spleen lymphocytes and oxidative stress

**Figure 4:**
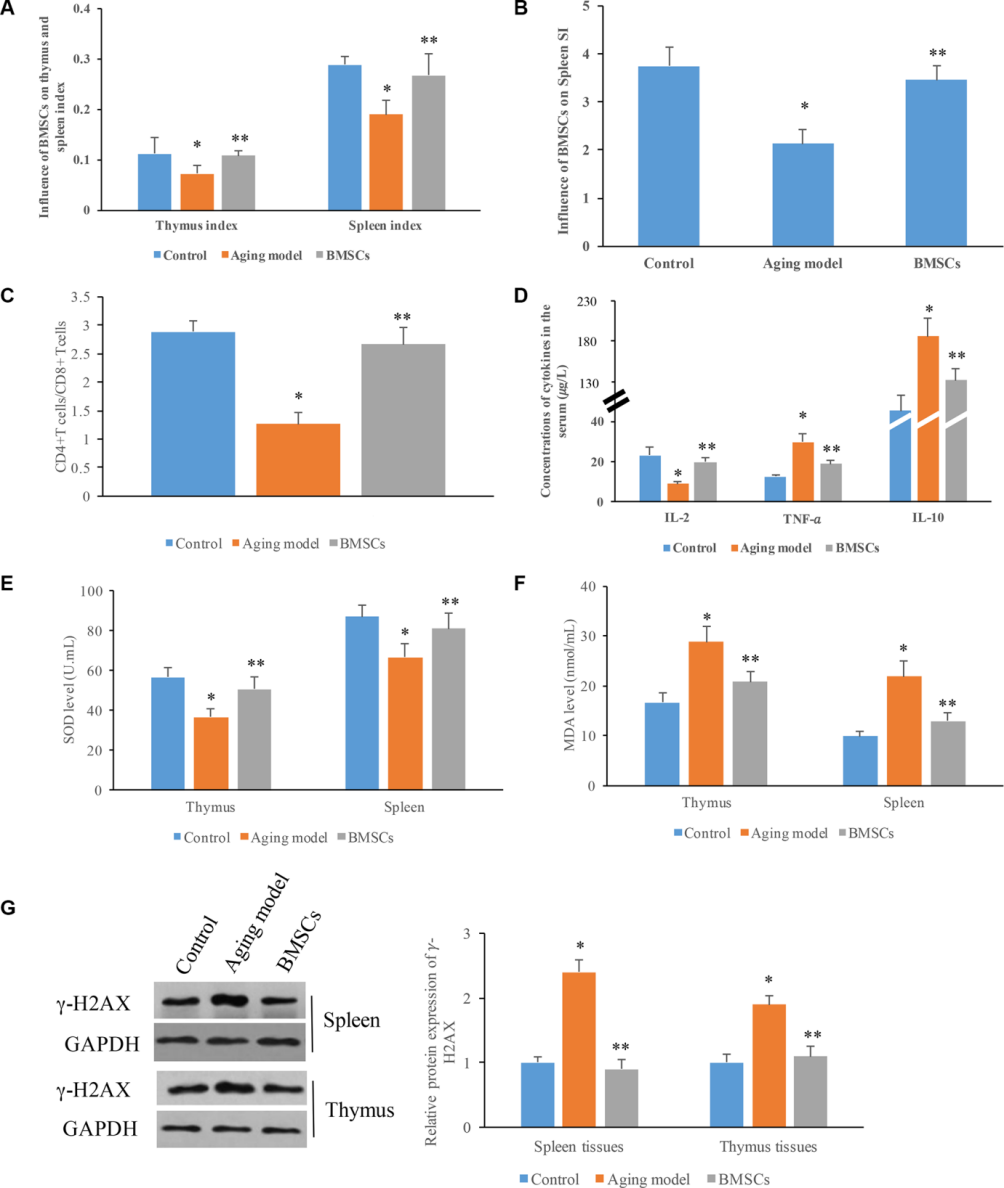
**Influence of BMSCs on the transformation function of spleen lymphocytes and oxidative stress.** (**A**) BMSCs significantly increased the thymus and spleen indexes; (**B**) BMSCs significantly increased the spleen SI; (**C**) Remarkable higher ration between CD4+ T cells and lower CD8+ T cells was achieved by BMSCs; (**D**) Influence of BMSCs on cytokines in the serum; (**E**) Influence of BMSCs on SOD levels in the tissues; (**F**) Influence of BMSCs on MDA levels in the tissues; (**G**) Influence of BMSCs on protein expression of γ-H2AX in the tissues. * P<0.05 compared with the control group; ** P<0.05 compared with the aging model group.

We demonstrated that the thymus and spleen indexes, and spleen SI were significantly decreased in the aging model group. However, BMSCs remarkably reversed the influence of D-galactose, and increased these items (Figure 4A and 4B). Meanwhile, significant lower ratio between CD4+T cells and CD8+T cells was found in the aging model group, it was markedly reversed by BMSCs (Figure 4C). In addition, we found that BMSCs could remarkably increase the levels of IL-2, and decrease the expression of IL-10 and TNF-*a* in the serum compared with aging model group (Figure 4D). Moreover, significant higher SOD and lower MDA in spleen and thymus tissues were found after treatment with BMSCs (Figure 4E and 4F). We also measured the protein expression of γ-H2AX, which is a marker of DNA damage. In the aging model, the level of γ-H2AX in the spleen and thymus tissues was increased significantly, but BMSCs suppressed γ-H2AX remarkably (Figure 4G).

### BMSCs improved the aging thymus and spleen by targeting the P21/PCNA signaling pathway

**Figure 5:**
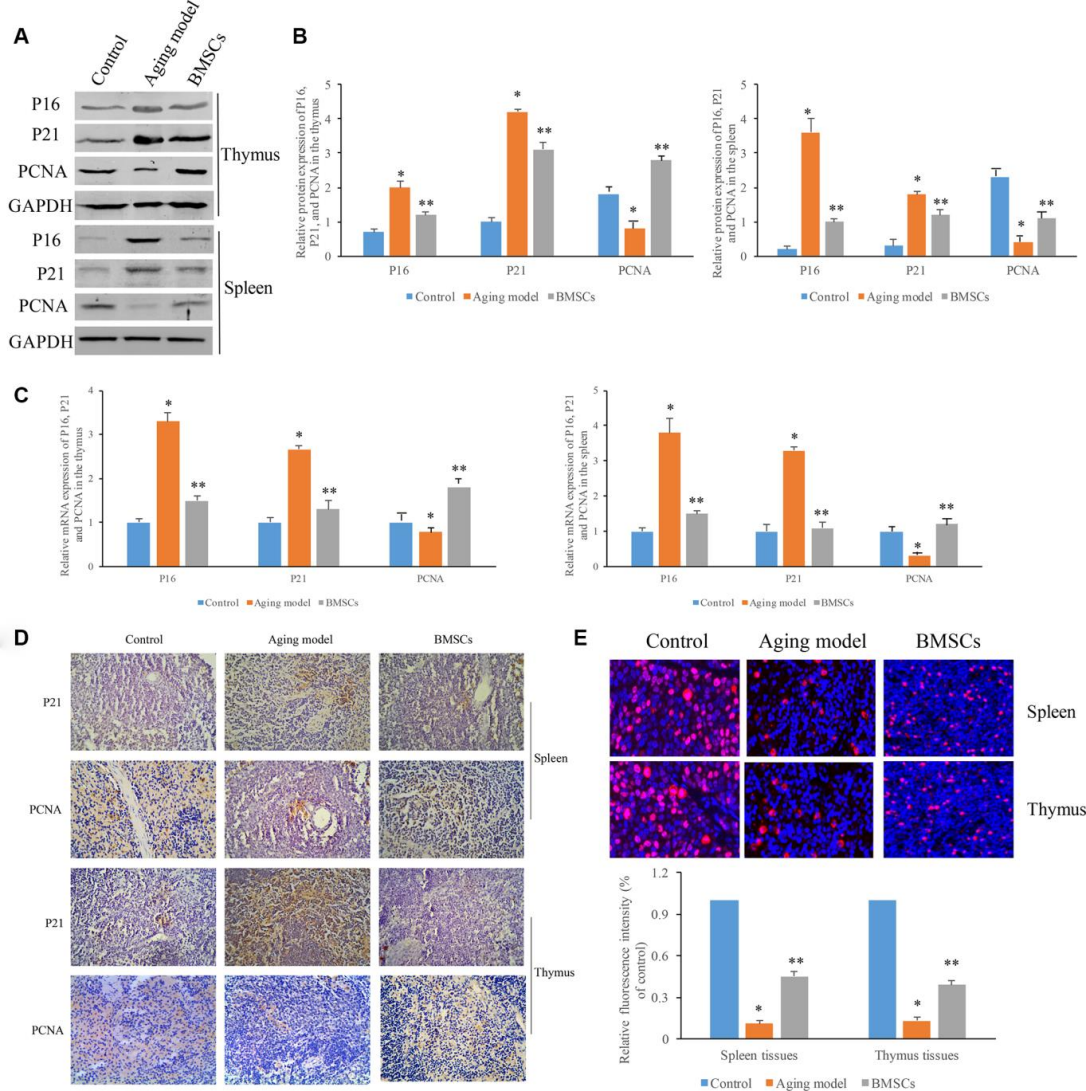
**BMSCs improved the aging thymus and spleen through targeting P21/PCNA signaling pathway.** (**A**) Protein expression of P16, P21, and PCNA were measured by western blotting; (**B**) Quantification analysis of protein expression of P16, P21, and PCNA; (**C**) Quantification analysis of mRNA expression of P16, P21, and PCNA; (**D**) Influence of BMSCs on P21 and PCNA in the tissues measured by immunohistochemical staining; (**E**) The proliferative status of the tissues (thymus and spleen) were analyzed by BrdU staining. * P<0.05 compared with the control group; ** P<0.05 compared with the aging model group.

To further investigate the possible mechanism BMSCs regulating aging thymus and spleen, we measured the influence of BMSCs on the expression of P21 and PCNA in the thymus and spleen tissues. We found that significant higher P21 and lower PCNA in both thymus and spleen tissues were observed in the aging model group. However, BMSCs could markedly reverse the effect of D-galactose inhibiting P21 expression and promoting PCNA (Figure 5A–5C). Therefore, BMSCs could suppress P21 and increase the expression of PCNA, which might be one of the mechanisms to reverse aging. Meanwhile, the effects of D-galactose and BMSCs on P21 was similar to that of P16 (Figure 5A–5C), which plays an important role in regulating cell cycle. The expression of P21 and PCNA in the tissues was also identified using immunohistochemical staining, and similar findings were observed compared with the results of western blotting and RT-PCR methods (Figure 5D). Meanwhile, the proliferative status of thymus and spleen were analyzed by BrdU staining. In the aging model, the proliferative status of thymus and spleen was suppressed, but BMSCs significantly reversed this trends (Figure 5E).

## DISCUSSION

Organ aging is a complex process involving many factors. Immune system plays an important role in body defense, self-stabilization and surveillance in vivo [24, 25]. The dysfunction of immune system could make the body vulnerable to the invasion of harmful factors. Current studies implicate that age-induced dysregulation of cytokine and hormone networks are closely linked with the loss of BMSCs [26, 27].

In the present study, we firstly isolated and identified BMSCs. Moreover, we demonstrated that the GFP-labeled BMSCs could be directionally delivered to thymus and spleen (Figure 2C and 2D). D-galactose has been commonly used to establish aging rat model for the reason that it could cause significant increase of free radicals triggering a chain reaction of lipid peroxidation, promote the level of lipid peroxide MDA aggravating the damage to cells, and suppress antioxidant enzyme SOD [28]. For the aging rats induced by D-galactose, the white pulp in the spleen was decreased remarkably, and the boundary between white pulp and splenic nodule was obscure. Meanwhile, thinner thymic cortex, bigger medulla, and less thymic lobule were found in the aging thymus tissues. Moreover, we observed more pyknosis, apoptosis, and adipose cells in the thymus tissues through TEM. However, treatment with BMSCs significantly improved the structure changes of spleen and thymus tissues.

The thymus index and spleen index of aging rats decreased markedly, and the decrease of thymus index suggested inhibition of cell immune function, thymocyte differentiation and proliferation ability [29]. The improvement of thymus index and spleen index caused by BMSCs (Figure 4A and 4B) was in agreement with the influence of BMSCs on structural changes of both thymus and spleen tissues.

Thymus is the place where T cells differentiate and develop. Because of thymus atrophy, the quantity and function of T cells will inevitably change [30]. Significant decrease of total number of T cells and increase of apoptotic T cells can be observed in the immune system of aging body. CD4+T cells were more easily to be induced to apoptosis than CD8+T cells, and the number decreased more obviously, which could lead to the inversion of the ratio between CD4+T cells and CD8+T cells [31, 32]. In this study, the ratio between CD4+T and CD8+T cells in thymus of aging model group was significantly lower than that of control group, indicating that the immune function of thymocytes decreased with aging. Compared to the aging model group, CD4+T cells/CD8+T cells increased remarkably in BMSCs treatment group. These findings indicated that BMSCs could slow down thymus atrophy, increase the number and function of T cells, and improve the immune function of body.

Aging has also been shown to dampen the secretion of IL-7 and IL-2, which are necessary survival cytokines for developing lymphocytes [33, 34]. IL-10 is amulti-potent immunoregulatory factor, and it exerts immunosuppressive effect via inhibiting the production of cytokines and destroying the balance of Th1/Th2 lymphocytes [35]. Our findings showed that aging could decrease the concentrations of IL-2, and increase IL-10 and TNF-*a* indicating thatthe immune function of thymus degrades with aging. However, remarkable increase of IL-2, and the suppression of IL-10 were found after treatment with BMSCs. These findings indicated that BMSCs could increase the function of T cells, and improve the immune function by influencing cytokines.

P21 is a broad-spectrum cyclin-dependent kinase inhibitor. The C-terminal of P21 has a unique binding site to PCNA, and P21 can inhibit the DNA replication activity of PCNA [16–19]. P16 and PCNA plays an important role in regulating cell cycle and promoting DNA replication. The expression of PCNA can reflect the proliferation of cells [36]. In the present study, we proved that BMSCs could markedly suppress the expression of P21, and promote the level of PCNA. BMSCs might improve the function of aging spleen and thymus through this mechanism. This study also showed that in the aging rat model induced by D-galactose, SOD activity decreased and MDA content increased markedly. However, BMSCs can effectively reduce the content of MDA, increase the activity of SOD. Therefore, BMSCs could improve anti-oxidative damage, and have a certain protective effect on aging rats. We noticed that the fluorescent labeling rate of BMSCs in the spleen tissues was lower, but the influence of BMSCs on the improvement of aging spleen, and the level changes of PCNA, P21, P16, SOD, and MDA in the spleen tissues was not affected.

## MATERIALS AND METHODS

### Laboratory animals and equipment

The SD male rats (8-10 weeks, 160-180 g) were purchased from experimental animal center of Fujian Medical University (animal number SYXK Min 2012-001), and all rats were divided into three groups depending on their age and weight: normal control group, aging model group induced by D-galactose and BMSCs treatment group. Five rats were in each group were guaranteed. The animals were treated in accordance with animal ethical standards during the experiment. All the animal experiments were authorized by the institutional Animal Care and Committee of Fujian Medical University.

### Reagents and instruments

Thermo Formo Cell Incubator (Thermo, Waltham, Mass USA), BS110S Electronic Taiping (Beijing Sartorius Electronics Co., Ltd, Beijing, China), OLYMPUS Inverted Microscope (Leica, Jena, Germany), COULTER Flow Cytometry (Coulter, USA), 721 Spectrophotometry (Shanghai Institute of Optoelectronics, Shanghai, China), H-600 Electron Microscope (Hitachi, Tokyo, Japan), Enzyme label detector (model 550, Tokyo, Japan), and D-galactose (Sigma, St. Louis, Mo, USA) were used in this study.

### Isolation and culture of bone marrow mesenchymal stem cells (BMSCs)

The rats were executed, and the femur and tibia were taken under aseptic conditions, and the bone marrow cavity was repeatedly washed to collect the cell suspension. After centrifugation (1000 r/min) for 3 min, the supernatant was discarded. Cells were resuspended in Dulbecco's Modified Eagle Medium (DMEM, Life Technologies, Carlsbad, USA) containing 10% FBS and then cultured in 37°C with 5% CO_2_. After about 2 weeks, the cells were passaged, and used for experiments.

### Phenotype identification of BMSCs

Passaged cells were adjusted to 2×10^5^ with PBS. Four random groups were added with different fluorescent labeling monoclonal antibodies (CD29, CD34, CD44, and CD45). Then flow cytometry was applied to detect cell surface marker. Mouse IgG1-FITC and IgG1-PE were simultaneously used as isotype antibody controls.

### Induction of BMSCs to steatoblast and osteoblasts

For the induction of steatoblast, 2×10^4^ BMSCs were seeded into 6-well plate, and 1 μmol/L dexamethasone, 10 mg/L insulin, 0.2 mmol/L indomethacin, and 10% FBS were added into DMEM. After 14 days’ induction, Oil red O staining was applied for identification.

For the induction of osteoblasts, 2×10^4^ BMSCs were seeded into 6-well plate, and osteoblast induction medium (10% FBS, 1.0×10^-8^ mol/L dexamethasone, 2.0×10^4^ mol/L ascorbic acid, 7.0×10^-3^ mol/L β-glycerophosphate) was added into DMEM. Change the medium every 3 days, after induction of 21 days, alizarin red staining was applied for identification.

### Establishment of D-galactose-induced aging animal model and treatment with BMSCs

Rats were injected subcutaneously with D-galactose daily through the neck and back at a dose of 400 mg/kg for 4 months. Animals were randomly divided into 3 different groups. The rats in the control group were treated with normal saline instead of D-galactose. The rats in the aging model group were administrated with D-galactose as described above, and treated with normal saline as the therapeutic method. The rats in the BMSCs group were administrated with D-galactose as described above, and then treated with allogeneic bone marrow-derived BMSCs (3×10^6^/time) through caudal vein injection once a week for 4 weeks. Then the tissues and serum were collected for additional experiments.

### GFP labeled BMSCs in the thymus and spleen tissues of aging rats

The passaged cells were added with adenovirus (EGFP-CMV) supernatant. Shake the plate slowly for 3 hours, and after another 48 hours’ culture, the infection was observed under a fluorescence microscope. Then 3×10^6^ BMSCs were infused into aging rats through tail vein. The rats were sacrificed 3 days later, and the thymus and spleen tissues were collected to make frozen sections, which were observed through a fluorescence microscope.

### Tissue preparation, HE staining and transmission electron microscopy (TEM)

Spleen and thymus tissues were separated after the sacrifice of rats using isoflurane inhalation. The tissues were firstly fixed using 10% formalin for 24 h. OCT compound was used for tissue embedding, and 10-μm thickness tissues were achieved using a frozen microtome. Five slides in each group were chosen for HE staining. Zeiss AxioVision was applied for capturing.

We further investigate morphological changes using transmission Electron Microscopy (TEM). After fixation with 1% glutaraldehyde, tissues were washed 3 times with PBS. Then fixation with 1% osmium tetroxide for 1 h, treatment with 1% uranyl acetate for 2 h, and embedding in epoxy resin were conducted subsequently. Finally, tissues were sectioned and observed using JEM1400 (Jeol, Japan).

### Immunohistochemical staining and BrdU staining

Tissues were separated after the sacrifice of rats, and fixed with 10% formalin for 24 h. OCT compound was used for tissue embedding, and 10-μm thickness tissues were achieved using a frozen microtome. Mouse anti-P21 antibody (ab80633, Abcam, Cambridge, UK) and mouse anti-PCNA antibody (ab29, Abcam, Cambridge, UK) were used in this study. Then, incubation with biotinylated secondary antibody and visualization with Vectastain Elite ABC HRP kit. Zeiss AxioVision was applied for visualizing.

For the BrdU staining, the BrdU was firstly dissolved into 10 mg/mL using PBS. The animals were treated with BrdU (100 mg/kg) through intraperitoneal injection. Tissue separation, embedding, section, and fixation were conducted as described above. Then, the sections were incubated with a mouse monoclonal anti-BrdU antibody (Abcam, Cambridge, UK). The proliferation data of tissues were analyzed by counting the BrdU stained positive cells (nuclei).

### Flow cytometry

The thymus tissues were grinded and 1640 culture solution was added. The cells were centrifuged at 3000 rpm for 5 min, and diluted to 10^6^ cells/ml. Then 10 μl of FITC-labeled CD4 antibody and PE-labeled CD8 antibody were added, and cells were incubated for 1 hour in the dark. 1% paraformaldehyde was used for fixing, and the ration between CD4+T cells and CD8+T cells in the thymus was measured by flow cytometry.

Cells were suspended using PBS, and divided into 4 groups. After incubation with FITC-labeled CD29, FITC-labeled CD34, PE-labeled CD44, and PE-CY5-labeled CD45, respectively, the cells were measured by flow cytometry.

### Western blotting

Tissues were grinded using liquid nitrogen for homogeneity firstly, and protein were lysed with RIPA lysis buffer containing 1% PMSF. After centrifugation at 13000 rpm/min for 10 min, the concentration of total protein in the supernatant was detected using BCA kit (CWBIO, Beijing, China). Same amount of protein from each group loaded and purified by 8% SDS-PAGE gels, and transfected to a polyvinylidene difluoride membrane. 5% nonfat milk was used for blocking, and gels were incubated with primary antibodies (1:800) overnight at 4°C, followed by incubation with second antibody (1:2000) in TBST at 37°C for 2 h. The protein bands were measured using ECL system. The antibodies used in this study were purchased from Abcam (Cambridge, UK). The specific information of antibodies was listed as follows: Rabbit Anti-p16 ARC antibody (ab51243); Rabbit Anti-p21 antibody (ab109520); Rabbit Anti-PCNA antibody (ab18197); Goat Anti-Rabbit IgG (ab205718).

### RNA isolation and RT-qPCR

Total RNA was extracted from tissues using TRIzol (Invitrogen, CA, USA), and 500 ng RNA was transcribed into cDNA using SuperScriptTM II Reverse Transcriptase (Invitrogen, CA, USA). SYBR Premix Ex TaqTM (Takara, Beijing, China) was used for determining cDNA. The primer sequences for PCR are as follows: P16: forward primer, 5'-GCGTTTGGAGAAGTGAGACAG-3', reverse primer, 5'-GAATACAATCAGCCCGGTTAAG-3'; P21: forward primer, 5'-TCCCTGCCCTGTAACTGTCTAAS-3', reverse primer, 5'-GCGTGGGCTCTTCCTATTACAT-3'; PCNA: forward primer, 5'-GATGTTCCTCTCGTTGTGGAG-3', reverse primer, 5'-CATTGCAGTTAAGAGCCTTCC-3'; GAPDH: forward primer, 5'-ACAACAGCCTCAAGATCATCAG-3', reverse primer, 5'-GGTCCACCACTGACACGTTG-3'. Data were analyzed by comparing cycle threshold values. The relative expression of target genes was analyzed using the 2^-∆∆Ct^ method.

### Detection of spleen index and thymus index

After the measurement of rat weight, rats were sacrificed. The spleen and thymus were isolated from rats and weighed immediately. Thymus index and spleen index were analyzed based on the following equation: Thymus index or spleen index = (weight of thymus or spleen)/body weight.

### Detection of cytokines, SOD, and MDA

The blood samples were collected through retro-orbital sinus puncture under isoflurane anesthesia. After centrifugation at 3000 r/min for 10 min, the serum was collected for detection. Then cytokines in the serum were detected by ELISA kits, respectively. After 4 weeks of MSCs treatment, the thymus and spleen tissues were collected. The levels of malondialdehyde (MDA) and the activity of superoxide dismutase (SOD) in the thymus and spleen were measured by thiobarbituric acid (TBA) method and xanthine oxidation (XTO) method, respectively.

### Statistical analysis

Statistical analysis was performed using SPSS 13.0 software, and the data were expressed as means ± SD. One-way analysis of variance (ANOVA) was used to compare the mean between groups, and the LSD method was used for comparison between groups. P value < 0.05 means statistically significant.
